# Influence of Grinding Process Parameters on the Three-Dimensional Surface Roughness of Silicon Carbide Particle-Reinforced Aluminum Matrix (SiCp/Al) Composites

**DOI:** 10.3390/ma19102070

**Published:** 2026-05-15

**Authors:** Zijun Li, Shaolei Wang, Yujing Zhao, Liying Zhang, Zhiwei Deng

**Affiliations:** 1 College of Mechanical Engineering, Guangdong Ocean University, Yangjiang 529500, China; emmadenni758@gmail.com (Z.L.); 18928585113@163.com (L.Z.); 15913137612@163.com (Z.D.); 2 College of Computer Science and Engineering, Guangdong Ocean University, Yangjiang 529500, China; yujing.zhao0823@gmail.com

**Keywords:** Sicp/Al composites, grinding process parameters, three-dimensional surface roughness, Pso-Bp neural network

## Abstract

Silicon carbide particle-reinforced aluminum matrix (SiCp/Al) composites are prone to surface defects during grinding owing to the heterogeneous deformation of the aluminum matrix and SiC particles, rendering conventional two-dimensional roughness parameters inadequate for precise surface characterization. In this study, three-dimensional surface roughness parameters were adopted to assess the ground surface quality of SiCp/Al composites. Orthogonal grinding experiments were carried out with four key process parameters (grinding wheel grit size, spindle speed, feed speed, and grinding depth), and the quantitative relationships between processing parameters and 3D roughness parameters, including arithmetical mean height (*Sa*), root mean square height (*Sq*), skewness (*Ssk*), kurtosis (*Sku*), surface bearing index (*Sbi*), core fluid retention index (*Sci*), and valley fluid retention index (*Svi*), were analyzed. The results reveal that the machined surface presents typical features including grooves from abrasive–matrix interaction, pits induced by SiC particle pull-out, scratches caused by dragged SiC particles, and tailing phenomena due to aluminum matrix melting under grinding heat. Grinding parameters exert distinct effects on surface topography: grinding wheel grit size shows the most significant influence on the *Sa* index, with its weight decreasing from 34% to 13% as grit becomes finer, while the combined influence weight of spindle speed, feed speed and grinding depth increases from 22% to 29%. Based on the comprehensive 3D roughness evaluation index, the optimal grinding parameter combination is determined as 320# grinding wheel, 4000 r/min spindle speed, 20 mm/min feed speed and 20 μm grinding depth. Additionally, the PSO-BP neural network achieves higher accuracy and better stability in predicting *Sa* and *Sci* than the conventional BP neural network.

## 1. Introduction

With the rapid development of industries such as automotive, aerospace, optical precision instruments and electronic packaging, metal matrix composite materials have completely replaced a variety of traditional materials in the high-tech field due to their superior performance, among which aluminum-based silicon carbide (SiCp/Al) composites are particularly prominent, offering high strength, wear resistance, corrosion resistance and a low expansion coefficient [1,2]. SiCp/Al is a composite material in which SiC reinforcing particles are introduced into an aluminum matrix, resulting in severe tool wear and more complex surface topography than that of conventional single-phase metals after machining. The measurement and characterization of the surface topography of parts can play a link function in the industry [3]. During processing, the presence of these reinforcing particles can induce various surface defects such as voids and cracks compromising surface quality and making SiCp/Al a challenging material to machine [4,5]. In the mold design and manufacturing of SiCp/Al composite materials, the measurement and characterization of part surface topography can play an important linking role in manufacturing and performance evaluation. On the one hand, the characteristics can be reflected to the processing link and the manufacturing process can be optimized; on the other hand, the quantifiable functions can be applied to the application link, and the service performance of parts can be evaluated to complete the closed cycle system of overall quality control [6]. Measurement and characterization of surface topography is an important means of quality control, and surface roughness is one of the important technical indicators for evaluating surface quality [7]. Traditional two-dimensional roughness parameters cannot fully capture the influence of deep voids and cracks, and the surface roughness results of different directions and positions are quite different, which cannot fully characterize the surface topography of the workpiece and cannot accurately reflect the service characteristics of the workpiece. For example, the surface lubricity of the inner and outer rings of precision bearings and the tightness of electronic packages [8]. Three-dimensional (3D) roughness parameters can reflect the surface shape and performance characteristics of the workpiece more comprehensively, accurately and intuitively from the horizontal and longitudinal aspects, which is suitable for characterizing the precision devices made from these composite materials [9,10], This area presents significant research opportunities.

Grinding has become a common machining method for SiCp/Al composites with its advantages of low cost, high material removal rate and good surface quality. Dong et al. investigated the removal mechanism of SiCp/Al materials involved in ultrasonic-assisted grinding (UAG) and conventional grinding (CG), examining grinding force, surface integrity, sub-surface damage, and grinding wheel wear [11]. Godino et al. established a correlation between grinding wheel topography, grinding wheel wear, and three-dimensional functional roughness parameters [12]. Duan et al. elucidated the formation mechanism of SiCp/Al surface defects and established a roughness extraction method based on the dual-tree complex wavelet transform (DT-CWT) [13]. Wang et al. carried out a rotating ultrasonic side grinding experiment on a 45% volume fraction SiCp/Al composite material, studying the effects of spindle speed, feed speed, cutting depth, and ultrasonic vibration amplitude on surface roughness [14]. Furthermore, Wang et al. selected typical 2D (*Ra* (arithmetic mean roughness), *Rp* (maximum peak height)) and 3D (*Sa* (arithmetical mean height), *Sq* (root mean square height)) surface roughness parameters to evaluate the influence of grinding parameters on the surface quality of aluminum alloys, detailing the three-dimensional surface topography [15]. Moreover, surface roughness is not merely a geometric descriptor, but an important surface integrity indicator that may influence wear behavior, contact performance, fatigue response, and corrosion susceptibility in service. The significance of comprehensive surface quality evaluation in predicting service performance has been broadly recognized across diverse engineering materials [16]. Therefore, improving the 3D surface topography of SiCp/Al composites is important for their functional reliability in precision applications.

Current research on SiCp/AI composite grinding mainly focuses on the coupled effects of various processing parameters on grinding force, surface roughness and surface topography [17,18,19]. Wang Jinfeng et al. used the Taguchi method to accurately predict the two-dimensional roughness parameters of SiCp/Al composites under different processing parameters [20]. Chuanmin Zhu et al. employed the Non-dominated Sorting Genetic Algorithm II (NSGA-II) to predict the two-dimensional roughness of SiCp/Al surfaces, achieving some improvement in grinding surface quality [21]. Zheng et al. effectively characterized the surface morphology and surface quality of SiCp/Al composites using the 3D parameter Scr, and effectively optimized the ultrasonic grinding process parameter using the NSGA-II [22]. Sun Hao et al. adopted neural network model to effectively improve the prediction accuracy of composite surface roughness [23]. Jinfeng Wang et al. used polynomial regression equation to predict the optimal surface roughness and its corresponding parameters, achieving good agreement with experimental results [24].

Existing prediction methods for workpiece surface roughness often rely on theoretical analysis, which may not fully capture the complex interactions of various machining factors. Experimental analysis requires extensive data collection and may lack generalization. However, artificial intelligence methods offer many advantages. For example, the backpropagation (BP) algorithm can obtain relevant grinding experimental data to establish a mapping between process parameters and surface roughness, enabling real-time prediction [25]. Peng Gu et al. used a particle swarm optimization-backpropagation (PSO-BP) neural network to predict the energy consumption in the grinding process of SiCp/Al composites, with high prediction accuracy [26].

Compared to previously published studies that predominantly rely on conventional two-dimensional roughness parameters (such as Ra) and standard analytical models, the value-added contribution of this research is twofold. First, this study extends the application of conventional 2D roughness evaluation toward 3D functional parameters in the context of SiCp/Al grinding. These 3D parameters (e.g., Sbi (surface bearing index), Sci (core fluid retention index)) volumetrically quantify actual component load-bearing capacity and core fluid retention—metrics that are critical for the anti-corrosion and tribological performance of precision SiCp/Al parts but remain entirely invisible to 2D traces. Second, traditional backpropagation networks are notoriously susceptible to becoming trapped in local minima when trained on the highly variable, stochastic datasets generated by composite grinding (due to random SiC particle fracture). To address this, the current research introduces a hybrid particle swarm optimization-backpropagation (PSO-BP) neural network. The PSO algorithm acts as a global metaheuristic regularizer, improving the robustness and stability of the prediction compared to conventional BP models. By integrating structured orthogonal experiments with this advanced hybrid prediction model, this study provides a practical and quantitative framework for optimizing the machining surface quality of high-volume fraction SiCp/Al composites.

## 2. Identification of 3D Parameters for 3D Surface Roughness Evaluation

The University of Birmingham, UK, laid the foundational work in three-dimensional roughness characterization, proposing a set of evaluation standards known as the Birmingham 14 parameters. These parameters encompass four main aspects of surface characteristics: amplitude information (*Sq*, *Ssk* (skewness), Sku (kurtosis), Sz (maximum height)), spatial information (Sds (density of summits), Str (texture aspect ratio), Sal (auto-correlation length), Std (texture direction)), complex information (S△q (root mean square gradient), Ssc (arithmetic mean summit curvature), Sdr (developed interfacial area ratio)), and functional information (Sbi, Sci, Svi (valley fluid retention index)).

Three-dimensional roughness parameters have many influences on surface properties, as shown in Table 1 below. Under different influence degrees, some three-dimensional roughness parameters were selected to characterize the surface of SiCp/Al composites.

As can be seen from Table 1, amplitude parameters have significant effects on all surface properties. The most commonly used three-dimensional roughness parameter Sa is an extension of the two-dimensional roughness parameter Ra. Therefore, amplitude parameters (Sa, Sq, Ssk, Sku) are selected to characterize the surface quality of SiCp/Al composites. The functional parameters reflect the surface micro-geometric characteristics and have a significant impact on all surface properties. The functional parameters (Sbi, Sci, Svi) are selected to characterize the workpiece surface. Although spatial and hybrid parameters theoretically influence certain surface functionalities, they were deliberately excluded from the prediction model due to specific physical and metrological constraints associated with SiCp/Al composites. From a physical perspective, SiCp/Al is a highly heterogeneous two-phase material. The random distribution of SiC particles and their stochastic pull-out mechanisms create highly irregular micro-defects. Consequently, spatial parameters (such as Sal and Str) primarily reflect the random phase distribution of the composite matrix rather than the deterministic kinematics of the grinding process. From a metrological perspective, hybrid parameters (such as Sdq and Sdr) are calculated based on the numerical spatial derivatives of the surface profile. Metrological studies have proven that these derivative-based parameters are extremely sensitive to high-frequency optical noise. During white-light interferometry, the steep edges of the pulled-out SiC pits inevitably induce high-frequency optical artifacts, which exponentially amplify the error in hybrid parameters. Therefore, relying exclusively on robust amplitude and functional parameters prevents the neural network from overfitting to metrological noise.

### 2.1. Amplitude Parameters

1. The amplitude parameter *Sa* (arithmetic mean deviation) refers to the arithmetic average distribution of the absolute height deviations within the measured surface roughness. The roughness is evaluated in the three-dimensional evaluation area, which has the characteristics of integrity and can effectively detect the height characteristics of the entire sample selection area.
2. The amplitude parameter *Sq* (root mean square deviation) is a statistical amplitude parameter, defined as the root mean square value of the measured surface roughness deviation from the reference standard in the sampling area, which can compare the micro-morphology changes of the parts before and after use.
3. The amplitude parameter *Ssk* (skewness) is the measurement of the symmetry of the surface deviation relative to the reference surface, and the measured roughness shape (concave and convex) tendency is judged by the *Ssk* value, reflecting the surface’s bearing properties.
4. Amplitude parameter *Sku* (kurtosis) is a measure of the kurtosis of the topography height distribution. Together with skew, it is proposed to describe the shape of the topography height distribution and identify the stability of the ground surface.

### 2.2. Function Parameters

1. The functional parameter *Sbi* (surface bearing index) is used to characterize the load-bearing performance of a surface. Generally, a flattened surface profile with truncated peaks yields a higher *Sbi* value, which corresponds to superior resistance to mechanical wear and contact deformation during service.
2. The functional parameter *Sci* (core fluid retention index) characterizes the capacity of the surface to retain fluids or lubricants in the central kinematic region. A larger *Sci* value indicates a greater void volume at the core, significantly enhancing the material’s anti-corrosion properties and hydrodynamic film maintenance.
3. The functional parameter *Svi* (valley fluid retention index) is used to characterize the fluid retention capacity specifically within the deep foundational valleys of the surface topography. A larger *Svi* value corresponds to enhanced deep-valley oil storage performance, which is vital for preventing catastrophic boundary lubrication failure in precision moving parts.

## 3. Test Content

### 3.1. Grinding Test Conditions

**Figure 1:**
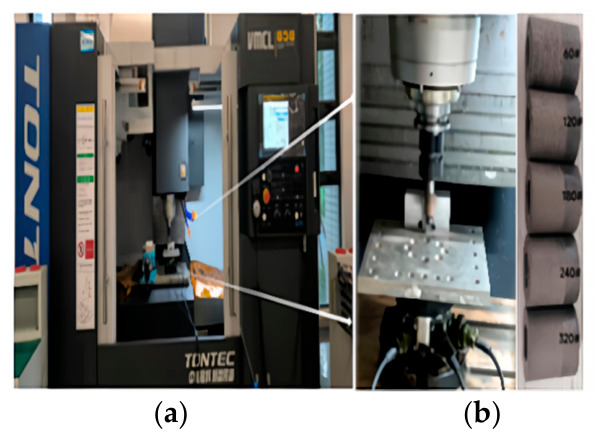

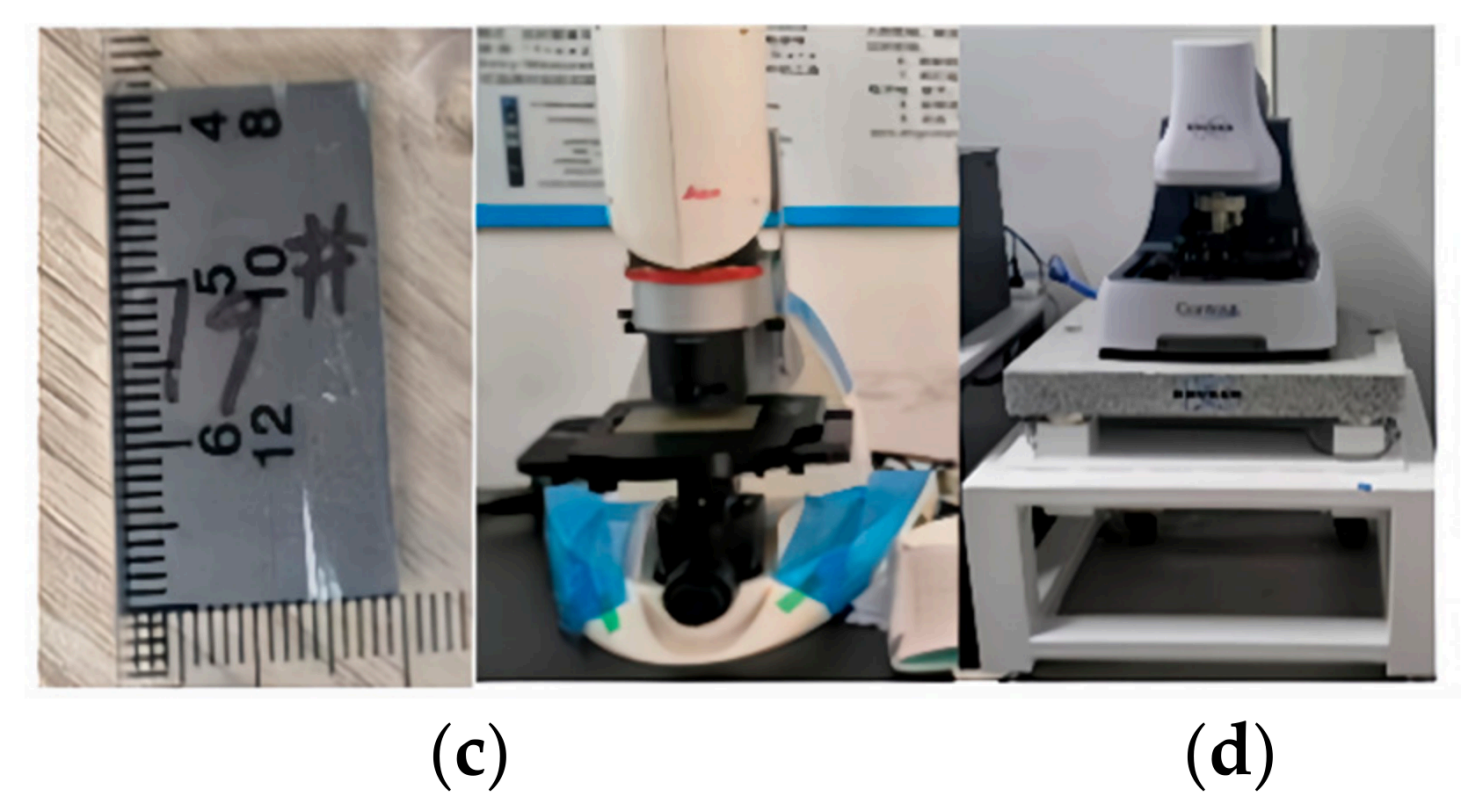
Test condition. (**a**) VCML850 vertical machining center. (**b**) Dynamometer and processing grinding wheel. (**c**) SiCp/Al workpiece. (**d**) Leica ultra-depth microscope and BRUKER white light interferometer.

The grinding test machine is VCML850 vertical machining center, which adopts dry grinding, as shown in Figure 1a. Resinous diamond grinding wheels with wheel counts of 60#, 120#, 180#, 240# and 320# were used for grinding, and the specifications were 20 mm in diameter × 20 mm in thickness × 10 mm in aperture. The surface of the specimen was ground by the side of the grinding tool, as shown in Figure 1b. SiCp/6092Al composite material (SiC reinforced particle volume fraction is 25%, average size is 35 μm) was cut into a 2 cm × 3.3 cm × 0.5 cm standard specimen, as shown in Figure 1c. The surface morphology and three-dimensional roughness parameters of scanned SiCp/Al composites were obtained by a Leica ultra-depth microscope and a BRUKER white light interferometer, as shown in Figure 1d.

The number of the grinding wheel (*Am*), the spindle speed (*Vs*), the feed speed (*Vf*) and the grinding depth (*ap*) as the four test factors are complicated. The orthogonal design can select the representative test data from the comprehensive test according to the orthogonality, which is an efficient, fast and economical experimental design method. As the level of each factor is shown in Table 2, the L25 (45) orthogonal table is selected for the grinding test. While a dataset of 25 experimental runs may appear small from the perspective of unstructured machine learning paradigms, the implementation of the Taguchi L25 ($5^{4}$) orthogonal array ensures maximum information density and absolute statistical representativeness. A conventional full-factorial experimental design investigating four factors across five levels would mathematically necessitate $5^{4}$ = 625 independent grinding runs, which is practically prohibitive due to the severe tool wear and high metrological costs associated with SiCp/Al composites. The fractional L25 array systematically compresses this vast parametric space while preserving the mutual mathematical independence among all columns, yielding a total of 24 statistical degrees of freedom. Because evaluating the independent main effects of the four grinding parameters requires only 16 degrees of freedom (4 × (5 − 1)), the 24 available degrees of freedom mathematically guarantee the adequacy of capturing the global variance and offer a practically efficient basis for preliminary regression modeling of the physical grinding process without relying on massive, redundant random sampling.

### 3.2. Introduction to Neural Network Structure

**Figure 2:**
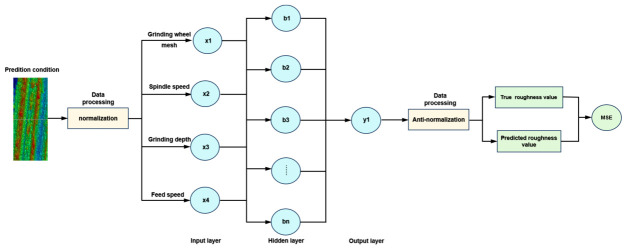
Prediction of 3D roughness structure by BP neural network.

A BP neural network is composed of an input layer, a hidden layer and an output layer. The core part is a backpropagation algorithm which iteratively optimizes the inter-layer parameters (weights and biases) to minimize the network’s error [27]. Because the number of hidden layers and neurons is difficult to determine in advance, overfitting may easily occur. Therefore, the BP neural network constructed selects a typical three-layer structure as shown in Figure 2, namely, input layer, hidden layer and output layer. The 25 datasets from the orthogonal experiments were partitioned into training and testing sets using an 8:2 ratio. The first 20 datasets formed the training set, while the remaining 5 constituted the test set. Before building the network model, in order to solve the comparability of data indicators, the data are normalized so that each value is in the range of (0, 1), and then the predicted results are reversibly normalized. The mean square error (*MSE*) value was selected to quantitatively evaluate the performance of the BP and PSO-BP prediction models. To avoid contingency, the training was repeated several times, and the predicted values and their average values were recorded and analyzed [28].

The adequacy of utilizing 20 samples for training and 5 for blind testing is firmly grounded in the topological simplicity of the problem space. Unlike complex computer vision models that suffer from the ‘curse of dimensionality’, the constructed backpropagation architecture models a highly constrained low-dimensional feature space, consisting of only four scalar input variables mapping to specific scalar output parameters (Sa, Sci). In such constrained engineering regression tasks, the requisite number of hidden neurons and weight parameters remains extremely small, allowing the network’s loss function to converge reliably using the structured orthogonal variance provided by the limited dataset. Furthermore, this specific 8:2 partitioning acts strictly as the outer holdout validation to assess the final model’s true generalization capability on completely unseen operational conditions. The test set was strictly excluded from the PSO optimization process and used only for final evaluation.

**Figure 3:**
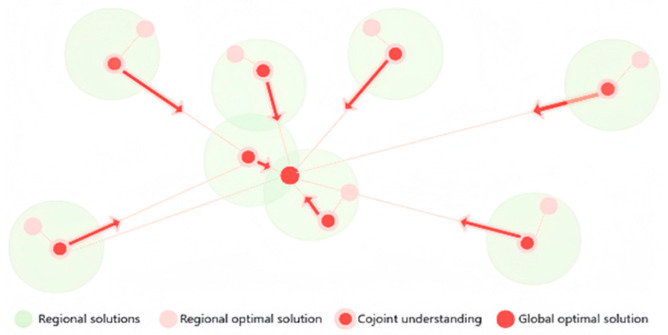
Optimization algorithm analysis.

The particle swarm optimization (PSO) algorithm takes the path of each particle’s trajectory as a potential solution and uses a fitness function to assess the quality of the solution. All particles move in accordance with the specified speed and direction in the feasible domain, exhibiting a tendency to converge towards the particle with the best-found solution. Through iterative exploration of the search space, the PSO algorithm identifies optimal solutions to the optimization problem (Figure 3).

### 3.3. PSO-BP Neural Network Structure

BP neural networks are well-established in both network theory and performance, offering advantages such as strong non-linear mapping capabilities and flexible network structure. However, BP neural networks also have several limitations: (1) slow convergence speed; (2) susceptibility to local minima; (3) lack of theoretical guidance for selecting the optimal number of hidden layers and neurons; and (4) limited generalization ability. The most common improvement measure is to optimize the BP neural network using PSO to reduce its probability of falling into a local optimum.

**Figure 4:**
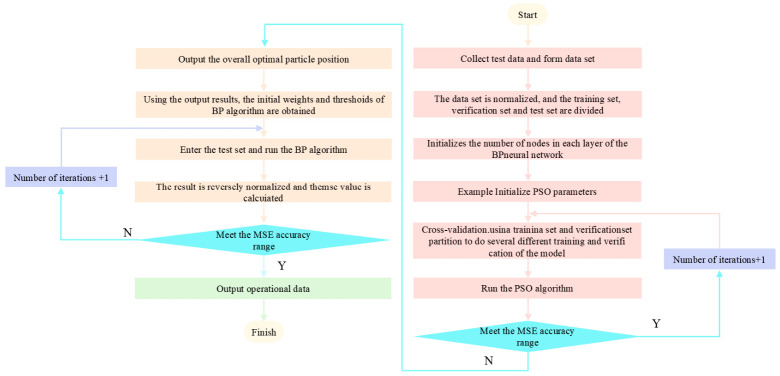
PSO optimizes BP algorithm flow.

The randomly given weight and threshold in the BP algorithm will affect the convergence speed of the prediction model and the selection of the global optimal value. The PSO algorithm is employed to determine suitable initial weights and biases for the BP algorithm, thereby improving its generalization performance. A fundamental vulnerability of standard BP neural networks when trained on small datasets is the rapid onset of overfitting. Random initialization of internal weights frequently causes gradient descent to become trapped in local minima, forcing the network to memorize training noise. While cross-validation is commonly used in random datasets, its benefit is less pronounced in this study due to the structured orthogonal sampling, which inherently minimizes sampling bias. To safeguard the generalization capability, the PSO algorithm was integrated to act as a powerful implicit regularizer. By conducting a global metaheuristic search strictly on the training set, the PSO algorithm assigns a near-global optimal set of initial weights to the BP network. This strategic initialization dramatically minimizes the requisite number of subsequent gradient descent iterations, fundamentally depriving the network of the iterative time necessary to overfit the training noise. Consequently, the PSO-BP hybrid framework ensures high stability and improved generalization performance when eventually exposed to the 5 out-of-sample testing datasets. The flowchart of PSO-BP algorithm is shown in Figure 4. The specific steps of optimizing the BP algorithm based on PSO are as follows.

(1) According to the experimental data, determine the number of input layer nodes *n*1, hidden layer nodes *n*2 and output layer nodes *n*3 of the BP neural network.
(2) Determine the dimension *D* of PSO according to the test requirements, *D* = *n*1 × *n*2 + *n*2 + *n*2 × *n*3 + *n*3.
(3) Provide training data, train the BP network structure, obtain appropriate weights and thresholds, and obtain the fitness of each particle.
(4) Determine the historical optimal value and global optimal value of each particle in the particle swarm, and update the velocity and position information of the particles. Determine whether the maximum number of iterations has been reached. If yes, terminate the iteration; otherwise, return to step (3) to continue the calculation.
(5) Use the weights and thresholds obtained by (4) as the corresponding parameter values of the BP algorithm.
(6) Execute the BP algorithm sequentially, meet the accuracy range, and end all algorithm operation.

## 4. Test Analysis

### 4.1. Specimen Surface Morphology

**Figure 5:**
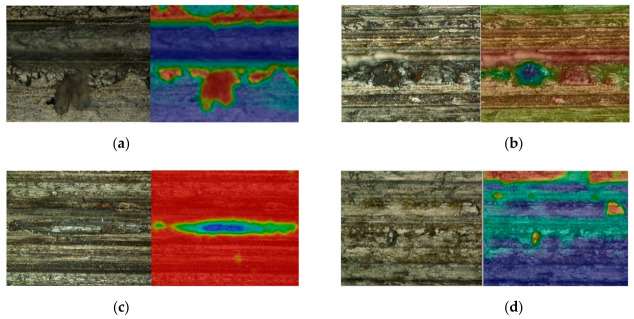
Surface defect. (**a**) Surface crack. (**b**) SiC particles form pits. (**c**) Scratch. (**d**) SiC particle protrusions.

In the process of grinding SiCp/Al, grinding wheel wear, crushing wear, etc., the aluminum matrix undergoes plastic deformation, SiC particles undergo brittle deformation, and combined deformation occurs under grinding heat and grinding forces in different directions. The surface morphology of the specimens has various defects, some of which are shown in Figure 5. The diamond abrasive tip is located in different positions of SiC particles, and has different degrees of brittle deformation, such as micro-crushing, fracture, crushing and falling off of SiC particles; the aluminum matrix has different plastic deformation under different conditions. The grinding heat generated during processing causes the aluminum matrix to melt and form the residual surface of molten slag. The surface temperature drops sharply and micro-cracks appear. The abrasive particles of the grinding wheel move across the aluminum matrix to create grooves.

**Figure 6:**
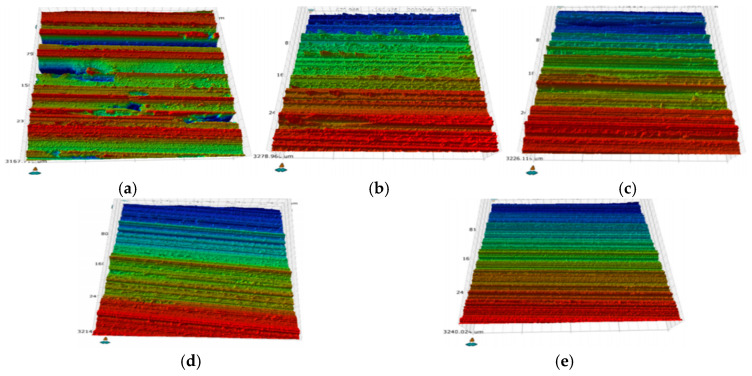
3D topography of SiCp/Al surface grinding with different grinding wheels. (**a**) 60#, (**b**) 120#, (**c**) 180#, (**d**) 240#, (**e**) 320#. The color bar denotes the surface height in micrometers (μm), where red corresponds to the highest elevation and blue corresponds to the lowest elevation.

The SiCp/Al grinding surface is formed under the interaction of diamond grinding particles, the aluminum matrix and SiC particles. The size of the grinding particles directly affects the surface morphology, and the grinding particles of different grinding wheel numbers have different effects on the surface. The results of the influence of different grinding numbers on SiCp/Al grinding surface are shown in Figure 6.

In Figure 6a, with a 60# grinding wheel, the larger diameter diamond abrasives primarily perform cutting with some sliding. This results in significant irregular pits on the surface due to the pulling out of SiC particles. In Figure 6b–d, with the grinding wheel 120#–240#, the thinner the grinding, the cutting and shearing actions become less pronounced, while the effect of the resin binder begins to enhance. This leads to a flattening of peaks and valleys, with grinding and groove marks becoming more evident. In Figure 6e (320# grinding wheel), the surface is mainly treated by slip brush, mainly resin binder sliding across the aluminum matrix. On the surface, clear scratches are visible. As the number of the grinding wheel increases (the finer the diamond grinding grain), the defective volume and the quantity of the surface of the test piece decreases, the grinding grain scratch becomes obvious, and the surface morphology improves, so the number of the grinding wheel significantly affects the surface morphology of SiCp/Al.

### 4.2. Three-Dimensional Roughness Parameter

#### 4.2.1. Influence of Spindle Speed on 3D Parameters of SiCp/Al Surface Under Different Wheel Counts

**Figure 7:**
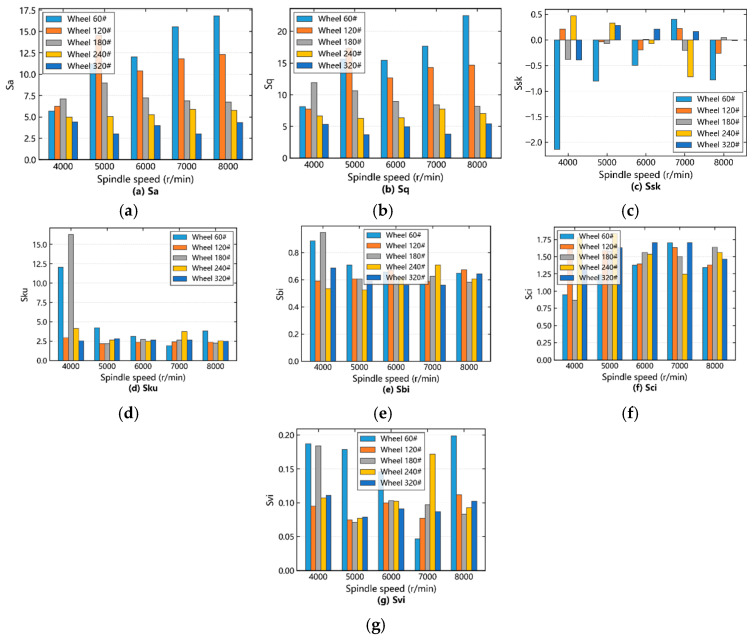
Influence of spindle speed on amplitude and functional parameters under varying grinding wheel grits: (**a**) *Sa*; (**b**) *Sq*; (**c**) *Ssk*; (**d**) *Sku*; (**e**) *Sbi*; (**f**) *Sci*; (**g**) *Svi*.

Using the 3D data of SiCp/Al surfaces, the change rules of 3D roughness parameters under different grinding process parameters are analyzed. In the above section, the grinding wheel number is an important factor obviously affecting the surface morphology, and the influence of the grinding parameters on 3D parameters under different grinding wheel numbers is analyzed from the numerical perspective. First, the influence of spindle speed on SiCp/Al surface amplitude and functional parameters under different numbers of grinding wheels was analyzed, as shown in Figure 7.

By analyzing the influence of different grinding wheel grits on the SiCp/Al surface amplitude parameters, (refer to the gray, purple, blue, orange, and green rectangles in Figure 7a–d), it can be observed that *Sa* and *Sq* tend to decrease with increasing grinding wheel grit size, and the functional characteristics of the surface are negatively correlated with *Sa* and *Sq*. The flatter the surface of the specimen is, the better the functional characteristics are, and the better the surface quality is. The distance between *Ssk* and 0 becomes smaller, *Sku* moves around 3, the surface height is symmetrically distributed, the sharp parts coexist, and the surface quality improves.

The influence of different grinding wheel counts on SiCp/Al surface functional parameters was analyzed. The gray, purple, blue, orange and green rectangles in Figure 7e–g were observed. As the number of grinding wheel counts increased, the influence of grinding wheel counts on functional parameters (*Sbi* and *Sci*) was less obvious than that of amplitude parameters (*Sa* and *Sq*). With the increase of wheel mesh number, the *Sbi* part increased, the *Sci* part decreased, the surface support was enhanced, the corrosion was not easy, the surface quality was better, the *Svi* range was 0.05–0.2, the oil storage performance was good, and the surface height of the specimen was in line with the normal distribution of Gaussian surfaces. Although the degree of change of *Ssk*, *Sku*, *Svi* also changes with the grinding process parameters, the value is generally changed around 0, 3, 0.11. According to the definition, it is necessary to have a certain understanding of the surface of its numerical characterization, and then there is no need to discuss further the change law of *Ssk*, *Sku*, *Svi*.

The influence of spindle speed on SiCp/Al surface functional parameters was analyzed. In Figure 7e,f, gray and green rectangles are observed when the grinding wheel was 60# and 320#. When the spindle speed was 4000–7000 r/min, the motion path of abrasive particles became shorter, the surface had large grooves along the feed direction, *Sbi* decreased, *Sci* increased, and the surface quality deteriorated. When the spindle speed is 7000–8000 r/min, the abrasive motion trajectory becomes longer, the ironing effect on the surface is enhanced, the *Sbi* is increased, the *Sci* is reduced, and the surface quality is improved. When the grinding wheel is 120#, the purple rectangle is observed. When the spindle speed is 4000–6000 r/min and 7000–8000 r/min, *Sbi* increases, *Sci* decreases, and the surface quality improves. When the spindle speed is 6000–7000 r/min, *Sbi* decreases, *Sci* increases, and surface quality deteriorates. When the spindle speed is 4000–5000 r/min and 7000–8000 r/min, *Sbi* decreases, *Sci* increases, and surface quality deteriorates. When the spindle speed is 5000–7000 r/min, *Sbi* increases, *Sci* decreases, and surface quality improves.

To physically explain the observed, and in some cases fluctuating, trends of Sbi and Sci as a function of spindle speed, a consistent physical model based on the competition between ‘SiC brittle pull-out’ and the ‘thermal-plastic ironing effect’ is proposed. The parameter Sbi characterizes the surface bearing capacity (flattened peaks), while Sci represents the core void volume for fluid retention (deep pits and grooves). At low-to-medium spindle speeds (e.g., 4000–7000 r/min for 60# and 320# wheels), the increasing kinetic impact of abrasives exacerbates the brittle fracture and pull-out of SiC particles without generating sufficient heat to melt the aluminum matrix. The dragged SiC fragments create deep scratches, which increases the core void volume (Sci increases) and reduces the flat bearing area (Sbi decreases). However, as the spindle speed enters the high-speed regime (7000–8000 r/min), extreme localized grinding heat is generated, causing the soft aluminum matrix to undergo severe thermal-plastic softening. In this regime, the ‘ironing and smearing effect’ dominates. The abrasive grains and the wheel bond iron the softened aluminum, smearing it over the previously formed SiC pull-out pits and grooves. This thermal-plastic smearing effectively fills the surface voids (Sci decreases) and flattens the profile peaks, drastically improving the load-bearing capacity (Sbi increases). The fluctuating trend observed with the 120# wheel (at 6000–7000 r/min) reflects a transitional zone where the intermediate abrasive size creates a highly sensitive competition between these two material removal mechanisms.

#### 4.2.2. Effect of Grinding Depth on 3D Parameters of SiCp/Al Surface Under Different Wheel Counts

**Figure 8:**
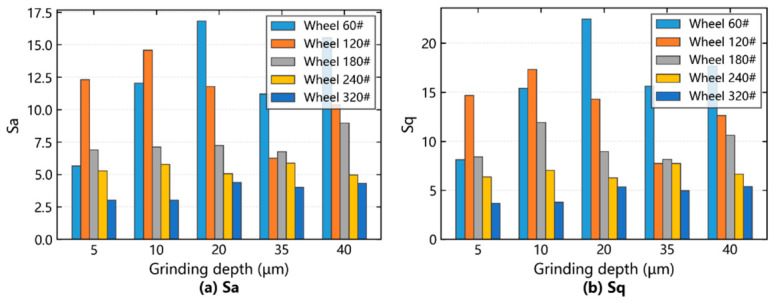

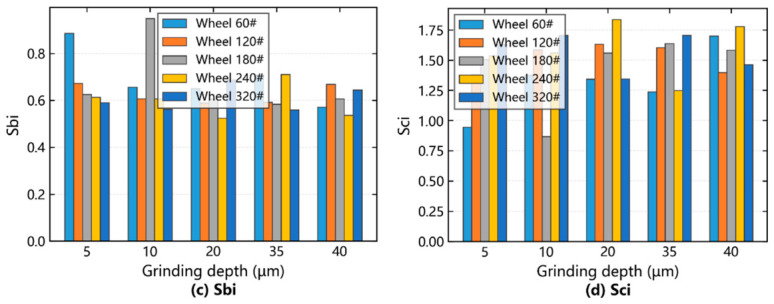
Effect of grinding depth on amplitude and functional parameters under varying wheel counts.

The variation rules of grinding depth on amplitude and functional parameters under different wheel counts were analyzed, as shown in Figure 8.

The influence of grinding depth on SiCp/Al surface amplitude parameters was analyzed. In Figure 8a,b, the gray rectangle was observed when the grinding wheel was 60#. When the grinding depth was 5 μm, 10 μm and 20 μm, the SiC particles changed from micro-crushing to brittle fracture and finally to complete brittle removal, and the surface quality of *Sa* and *Sq* deteriorated during the increasing stage. The average size of SiC particles was 35 μm. When the grinding depth is 35 μm, more SiC particles are pulled out, *Sa* and *Sq* decrease, and the surface quality improves. When the grinding depth is 40 μm, the SiC particles are micro-broken, the surface quality deteriorates, and the *Sa* and *Sq* increase, but they are not higher than the *Sa* and *Sq* at the grinding depth of 20 μm. When the grinding wheel is 120#, observe the purple rectangle. When the grinding depth is 5–10 μm, SiC particles are broken more, *Sa* and *Sq* increase, and the surface quality deteriorates. When the grinding depth is 10–35 μm, SiC particles are mostly broken and pulled out, *Sa* and *Sq* decrease, and the surface quality improves. When the grinding depth is 35–40 μm, the amplitude parameter changes the same as the grinding wheel 60#. When the grinding wheel is 180#–320#, the blue, orange and green rectangles are observed, the abrasive grit becomes finer, the surface is mainly scratched, the SiC particles are cut more, and the amplitude parameter changes are small.

The influence of grinding depth on SiCp/Al surface functional parameters was analyzed. In Figure 8c,d, the gray rectangle was observed when the grinding wheel was 60#. When the grinding depth was 5–10 μm and 35–40 μm, SiC particles were broken more, *Sbi* decreased, *Sci* increased, and surface quality deteriorated. When the grinding depth is 10–35 μm, *Sbi* increases, *Sci* decreases, and surface quality improves. When the grinding wheel is 120#, observe the purple rectangle. When the grinding depth is 5–20 μm, *Sbi* decreases, *Sci* increases, and the surface quality deteriorates, but when the grinding depth is 20–40 μm, *Sbi* increases, *Sci* decreases, and surface quality improves. When the grinding wheel is 180#, observe the blue rectangle. When the grinding depth is 5–10 μm and 35–40 μm, *Sbi* increases, *Sci* decreases, and the surface quality improves, but when the grinding depth is 10–35 μm, *Sbi* decreases, *Sci* increases, and surface quality deteriorates. When the grinding wheel is 240#, the orange rectangle is observed. When the grinding depth is 5–20 μm and 35–40 μm, *Sbi* decreases, *Sci* increases, and surface quality deteriorates, butwhen the grinding depth is 20–35 μm, *Sbi* increases, *Sci* decreases, and surface quality improves. When the grinding wheel is 320#, the green rectangle is observed. When the grinding depth is 5–10 μm and 20–35 μm, *Sbi* decreases, *Sci* increases, and surface quality deteriorates, but when the grinding depth is 10–20 μm and 35–40 μm, *Sbi* increases, *Sci* decreases, and surface quality improves.

#### 4.2.3. Influence of Feed Speed on 3D Parameters of SiCp/Al Surface Under Different Wheel Counts

**Figure 9:**
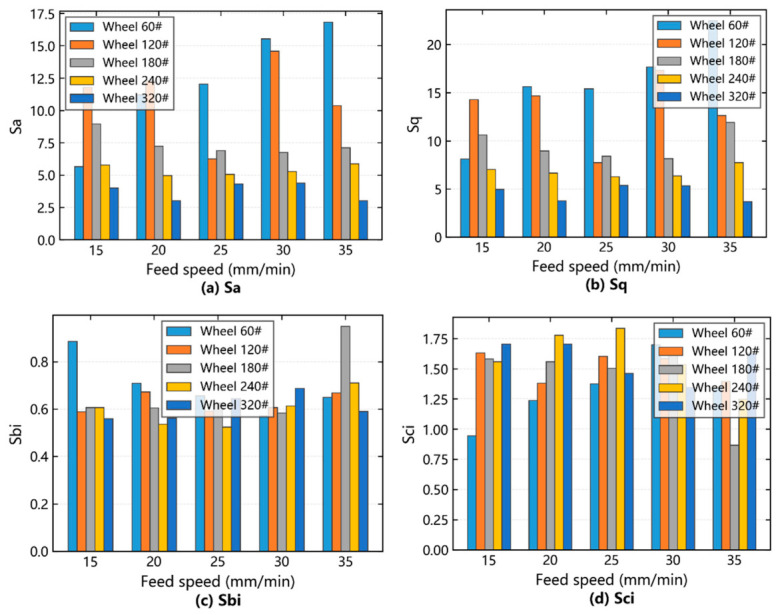
Influence of feed speed on amplitude and functional parameters under different wheel mesh numbers.

The variations in amplitude and functional parameters of the SiCp/Al surface due to feed speed under different grinding wheel grits were analyzed, as shown in Figure 9.

The influence of feed speed on SiCp/Al surface amplitude parameters was analyzed. In Figure 9a,b, when the grinding wheel was 60#, the gray rectangle was observed. When the feed speed was 15–20 mm/min and 25–30 mm/min, the interaction between abrasive particles was small, the grooves were deep and wide, *Sa* and *Sq* were increased, and the surface quality was poor. Conversely, at feed speeds of 20–25 mm/min and 30–35 mm/min, the abrasive particles act on the surface for a long time, the tracks between different abrasive particles are superimposed, the surface grooves are shallow and narrow, the multi-fine network is convex peaks and pits, *Sa* and *Sq* decrease, and the surface quality is improved. When the grinding wheel is 120# processing, observe the purple rectangle. When the feed speed is 15–20 mm/min, the abrasive action time on SiC particles is short, SiC particles are broken more, *Sa* and *Sq* increase, and the surface quality is degraded. When the feed speed is 20–35 mm/min, multiple abrasive particles act on SiC particles, SiC particles are cut more, *Sa* and *Sq* decrease, and the surface quality improves. When the grinding wheel is 180#–320#, observe the blue, orange and green rectangles; the feed speed increases, and the amplitude parameter changes gradually decrease.

The influence of feed speed on SiCp/Al surface functional parameters was analyzed. In Figure 9c,d, the gray rectangle was observed when the grinding wheel was 60#. When the feed speed was 15–20 mm/min and 25–30 mm/min, the interaction between abrasive particles was small, *Sbi* decreased, *Sci* increased, and the surface quality was poor. When the feed speed is 20–25 mm/min and 30–35 mm/min, the abrasive particles act on it for a longer time, the grooves on the machining surface are shallow and narrow, the *Sbi* increases, the *Sci* decreases, and the surface quality is better. When the grinding wheel is 120#, observe the purple rectangle; when the feed speed is 15–25 mm/min, *Sbi* increases, *Sci* decreases, and the surface quality improves, but when the feed speed is 25–35 mm/min, *Sbi* decreases, *Sci* increases, and surface quality deteriorates. When the grinding wheel is 180# and 240#, observe the blue and orange rectangles. When the feed speed is 15–20 mm/min, when the grinding wheel is 180#, *Sbi* decreases, *Sci* increases, and the surface quality deteriorates, while for the 240# wheel at feed speeds of 20–30 mm/min, *Sbi* increases, *Sci* decreases, and surface quality improves. When the feed speed is 30–35 mm/min, *Sbi* decreases, *Sci* increases, and surface quality deteriorates. With a grinding wheel of 320#, observe the green rectangle; when the feed speed is 15–20 mm/min and 25–35 mm/min, *Sbi* increases, *Sci* decreases, and the surface quality is better, but when the feed speed is 20–25 mm/min, *Sbi* decreases, *Sci* increases, and surface quality deteriorates.

### 4.3. Effects of Different Grinding Processes on 3D Parameters

From the numerical point of view, the influence of each grinding process parameter on the three-dimensional parameters under different grinding wheel counts was directly analyzed. Subsequently, the influence weight of grinding process parameters on the three-dimensional parameters was analyzed based on the range analysis; on the other hand, the optimal combination of factors corresponding to the comprehensive index of three-dimensional parameters was found based on factor analysis, and the influence of different grinding processes on the three-dimensional roughness parameters was studied and analyzed.

#### 4.3.1. The Influence Weight of Grinding Process Parameters on 3D Parameters

The range analysis is the most commonly used method to analyze orthogonal test data, where the range value indicates the degree of influence of each factor on the test index, and can be used to judge the primary and secondary order of processing factors. As can be seen from the analysis content in the above section, it is not necessary to conduct in-depth analysis on parameters *Ssk*, *Sku* and *Svi*, and the variation range of parameters *Sbi* and *Sci* is too small. Therefore, this paper analyzes the influence weight index w (weight) of grinding process parameters on amplitude parameters *Sa* and *Sq* based on the quantitative index of range analysis, namely:

(1) $$w_{\mathrm{nij}}=\frac{\bar{k_{\mathrm{nij}}}}{\sum_{j=1}^{4}\bar{k_{\mathrm{nij}}}}$$

where $w_{\mathrm{nij}}$ is the influence weight of factor i on the three-dimensional parameter index in the experiment of group n; n is the number of factors; and $\bar{k_{\mathrm{nij}}}$ is the average value of test data under the level j factor in group n.

**Figure 10:**
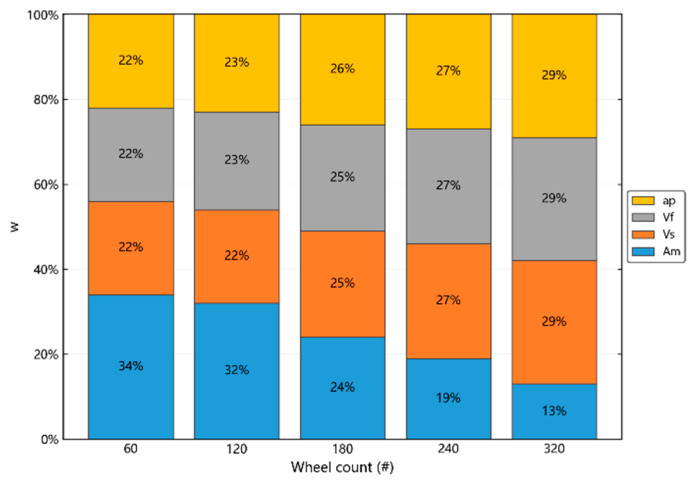
The effect of each grinding process parameter on *Sa* w under different wheel counts.

According to the orthogonal experiment scheme, the weights of various factors and levels on the amplitude parameters *Sa* and *Sq* in each group of tests were calculated. The results showed that although the grinding process parameters had slightly different w of the amplitude parameters *Sa* and *Sq* at different levels, the w of *Sa* and *Sq* were very similar. In addition, because the grinding wheel counts are significantly different from other process parameters in terms of amplitude parameters *Sa* and *Sq* w at different levels, the mean values of each grinding process parameter to amplitude parameter *Sa* under different grinding wheel counts are calculated, as shown in Figure 10.

With the increase of the number of grinding wheels, observe the pink rectangle in Figure 10, and the number of grinding wheels decreases from 34% to 13% of the amplitude parameter *Sa*; observe the yellow, green and blue rectangles in Figure 10, and the spindle speed, feed speed and grinding depth gradually increase from the initial 22% to 29%. When the grinding wheel is 60# and 120#, compared with other grinding process parameters, the number of grinding wheel eyes of the pink rectangle has the largest effect on the amplitude parameter *Sa*, and when the grinding wheel is 180#, 240# and 320#, the number of grinding wheel eyes has the smallest effect on the amplitude parameter. When the grinding wheel is 180#, the grinding process parameters are more uniform to the amplitude parameters, and the value is about 25%.

#### 4.3.2. Comprehensive Index of 3D Parameters

Each 3D parameter has different meanings, and the optimal combination of processing parameters for each group of orthogonal tests is also different. A comprehensive parameter index is needed to characterize each 3D parameter. The factor analysis method extracts objective weights based on intrinsic information enrichment. The calculated Kaiser–Meyer–Olkin (KMO) and Bartlett’s test results are listed in Table 3, and the extracted component weights after rotation are presented in Table 4.

The comprehensive index (CI) physically acts as a holistic ‘surface integrity score’. In machining, individual 3D parameters often present multi-objective conflicts (e.g., minimizing amplitude *Sa* might inadvertently degrade the functional fluid retention *Sci*). The CI mathematically integrates these distinct amplitude and functional dimensions into a single scalar, allowing for the identification of a global optimal grinding state. To avoid subjective human bias, factor analysis was employed to objectively extract the weights (*Wi*) based on intrinsic variance. As shown in Table 3, the Kaiser–Meyer–Olkin (KMO) measure is 0.51. Although the KMO value is relatively low, it still satisfies the minimum acceptable threshold for factor analysis. It is a predictable and logical mathematical consequence of our rigorous experimental design. Specifically, the low KMO value reflects the fact that the 25 samples were generated via a highly uncorrelated L25 orthogonal array, and the selected 3D parameters were deliberately chosen to represent independent physical traits (minimizing redundant collinearity). Despite the inherently low common variance induced by this orthogonal design, the Bartlett’s test of sphericity remains statistically significant (*p* < 0.05), validating that the correlation matrix is strictly adequate for extracting objective weights.

**Figure 11:**
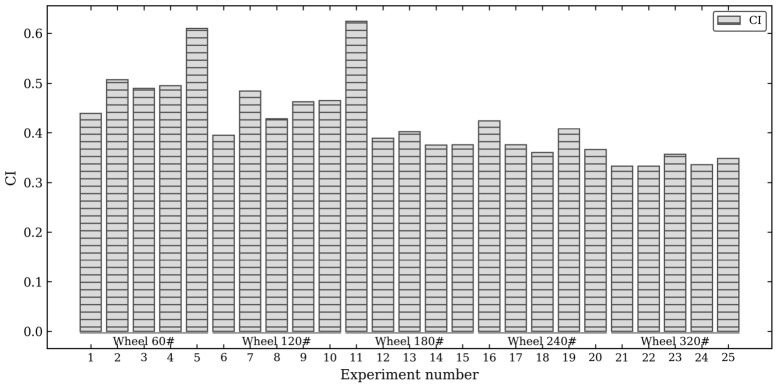
Comprehensive index diagram of 3D parameters.

The 3D parameters of different attributes, orders of magnitude and units are processed with unified transformation methods and formulas, so that these indicators are in the same order, and these indicators are pulled to a unified baseline for comprehensive weighted evaluation operation. After processing the amplitude parameters (*Sa*, *Sq*, *Ssk*, *Sku*) and functional parameters (*Sbi*, *Sci*, *Svi*), the 3D parameter comprehensive index CI is shown in Formula (2), and the results after calculation according to the formula are shown in Figure 11.

(2) $$\mathrm{CI}=\sum_{i=1}^{7}\frac{X_{\mathrm{ni}}-X_{\mathrm{niMIN}}}{X_{\mathrm{niMAX}}-X_{\mathrm{niMIN}}}\times W_{i}$$

where $X_{\mathrm{ni}}$ is the i th three-dimensional parameter of the n group test, $X_{\mathrm{niMAX}}$ and $X_{\mathrm{niMIN}}$ are the minimum and maximum values of the i th three-dimensional parameter of the n group test, and Wi is the weight of the i th three-dimensional parameter in Table 4.

**Figure 12:**
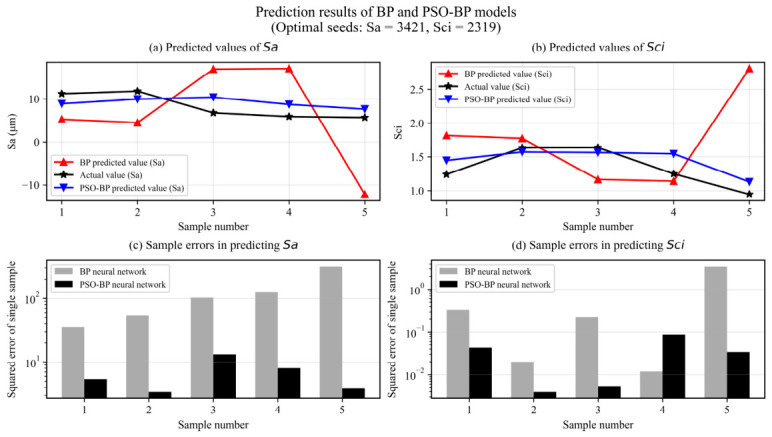
Comparison of the prediction results of the BP and PSO-BP models for *Sa* and *Sci*, together with the corresponding single-sample squared errors on the test set. The plotted results correspond to the optimal model outputs obtained under the selected random initialization.

According to the pink rectangle observed in Figure 11, the larger the number of the wheel mesh, the better the surface quality. Meanwhile, the CI values corresponding to 120#, 240# and 320# of the wheel did not change much, and the surface quality of the specimen was relatively stable. The test number corresponding to the minimum CI in Figure 12 is group 21, which is the combination of optimal grinding processing parameters: the number of grinding wheels is 320#, the spindle speed is 4000 r/min, the feed speed is 20 mm/min, and the grinding depth is 20 μm.

### 4.4. Analysis of Neural Network Prediction Results

The results of the 3D parameters under different grinding processes show that the variation of *Sa* in amplitude parameters is similar to *Sq*, and the values of *Ssk* and *Sku* are not suitable for prediction. The variation of *Sbi* in functional parameters is opposite to *Sci*, and the value of *Svi* is not suitable for prediction. The most commonly used three-dimensional parameter in previous studies is *Sa*, and there are many studies on the corrosive function of specimens, so the neural network is selected to predict the three-dimensional parameters *Sa* and *Sci*.

Because the grinding wheel grit size significantly influences the surface in other grinding processes, one dataset was randomly selected from each grinding wheel mesh number in five groups of test samples, and suitable model configurations were established after repeated training through the BP and PSO-BP neural network model training sample data. For example, the number of hidden nodes, the number of iterations, the learning rate, the activation function, the *MSE* accuracy range, etc., the predicted value, the true value and the *MSE* value of the three-dimensional parameters of the network model were obtained.

The BP and PSO-BP neural network models were trained to obtain the predicted values, true values and *MSE* values of three-dimensional parameters *Sa* and *Sci*, as shown in Figure 12.

It can be seen from Figure 12 that the predictions of 3D roughness parameters *Sa* and *Sci* obtained by the PSO-BP model are significantly closer to the experimental values than those obtained by the BP network. As indicated by the marked regions in the figure, the mean squared error (MSE) of the PSO-BP network is drastically lower than that of the BP network.

The quantitative results show that the test MSE values of the BP network for *Sa* and *Sci* were 126.9568 and 0.8110, respectively. In contrast, the PSO-BP model reduces the MSE to 6.7657 (for *Sa*) and 0.0347 (for *Sci*), corresponding to error reductions of 94.7% and 95.7%.

The BP neural network establishes a nonlinear mapping between grinding parameters (wheel mesh size Am, spindle speed Vs, feed speed Vf, grinding depth ap) and 3D roughness parameters *Sa* and *Sci*. However, random initialization of weights and biases tends to cause large fluctuations and poor generalization.

The PSO algorithm optimizes the initial weights and biases of the BP network to avoid local optima and enhance stability. With optimized initial parameters (optimal random seeds: 3421 for *Sa* prediction, 2319 for *Sci* prediction), the PSO-BP model achieves more robust and accurate prediction, which effectively suppresses the interference of random factors in the grinding process. Furthermore, because conducting multiple physical repetitions for all 25 severe grinding runs is economically and practically prohibitive due to extreme diamond tool wear when machining SiCp/Al, the model-based prediction uncertainty was quantified using the out-of-sample root mean square error (RMSE) derived from the blind test set. The RMSE serves as an unbiased statistical estimator of the measurement and process noise. For the identified optimal parameter combination, the 1σ prediction uncertainty ranges are established at approximately ±2.60 for the amplitude parameter *Sa* (derived from MSE = 6.7657) and ±0.186 for the functional parameter *Sci* (derived from MSE = 0.0347). These narrowly bounded uncertainty intervals confirm that the established optimal grinding state is highly stable against the inherent stochasticity of SiC particle pull-out.

## 5. Conclusions

In this study, SiCp/Al composites were processed with different grinding process parameters. Based on the experimental data and microscopic observations, the following five conclusions are drawn.

(1) When different diamond grinding grain diameter is processed, diamond grinding grain, resin binding agent, aluminum matrix, SiC particles cut SiCp/Al surface, and the processing traces are different. The number of the grinding wheel obviously affects the surface morphology of SiCp/Al. When the number of the grinding wheel increases, the finer the diamond grinding grain, the better the surface morphology of the specimen.
(2) The influence of grinding process parameters on the amplitude parameters (*Sa*, *Sq*) is more significant than the functional parameters (*Sbi*, *Sci*). A finer grinding wheel grit size leads to better surface quality of the specimen; the spindle speed will change due to the grinding particle motion track and pressing effect; SiC particles can be removed due to the grinding depth; and the feeding speed affects the interaction time between the grinding particles and thus affects the surface quality.
(3) The grinding process parameters have different weights on the amplitude parameters *Sa* and *Sq* at different levels. When the grinding wheel is 60# and 120#, the influence weight of the grinding wheel mesh on the amplitude parameter *Sa* is prominent; with the increase of the grinding wheel mesh, the influence weight on the amplitude parameter *Sa* decreases from 34% to 13%. When the grinding wheel is 180#, 240# and 320#, the influence weight of the grinding wheel is reduced from 34% to 13%. Spindle speed, feed speed, grinding depth begin to become significant factors, from the initial weight of 22% gradually increased to 29%. When the grinding wheel is 180#, the influence weight of each grinding process parameter on the amplitude parameter *Sa* is uniform.
(4) The calculated comprehensive index (CI) indicates that the larger the number of grinding wheel mesh, the better the surface quality. The three-dimensional parameter index data corresponding to the optimal grinding processing parameter combination are: grinding wheel mesh *Am* = 320#, spindle speed *Vs* = 4000 r/min, feed speed *Vf* = 20 mm/min, and grinding depth *ap* = 20 μm. Based on the robust validation of the PSO-BP model, the prediction uncertainty ranges for this optimal surface quality are bounded within ±2.60 for *Sa* and ±0.186 for *Sci*.
(5) BP and PSO-BP neural network models were established to predict the 3D roughness parameters *Sa* and *Sci*. Quantitative validation demonstrates that the PSO-BP network demonstrates significantly improved performance compared to the conventional BP model. The PSO-BP model reduced the mean square error (MSE) for the amplitude parameter *Sa* by 94.7% (dropping from 126.9568 to 6.7657) and for the functional parameter *Sci* by 95.7% (dropping from 0.8110 to 0.0347). The algorithmic optimization provided by PSO effectively filters the stochastic noise inherent in SiC particle fracture, showing improved prediction accuracy and stability for composite machining.

## Figures and Tables

**Table 1:** The impact of three-dimensional roughness parameters on surface properties.

Surface Property	Amplitude Parameter	Spatial Parameter	Functional Parameter	Mixed Parameter
Supporting performance	▲	▲	▲	△
Leakproofness	▲	△	▲	▲
Abrasion resistance	▲	▲	▲	▲
Contact stiffness	▲	△	▲	△
Lubricating property	▲	▲	▲	△
Electrical/thermal conductivity	▲	▲	▲	▲
Anti-scratch	▲	_∘_	▲	▲
Weld and paste	▲	_∘_	▲	△
Decorative	▲	△	▲	△
Modificability	▲	▲	▲	△
Fatigue strength	▲		▲	_∘_

Note: ▲ indicates significant impact, △ indicates moderate impact, _∘_ indicates small impact or selected with the environment.

**Table 2:** Orthogonal test factor levels.

Factor Level	Am/(#)	Vs/(r·min^−1^)	Vf/(mm·min^−1^)	ap/(μm)
1	60	4000	15	5
2	120	5000	20	10
3	180	6000	25	20
4	240	7000	30	35
5	320	8000	35	40

**Table 3:** KMO and Bartlett’s test of sphericity.

KMO value		0.51
Bartlett sphericity test	Approximate chi-square	336.455
	df	21
	*p*-value	0

**Table 4:** Factor analysis weight results for 3D roughness parameters.

Name	Factor 1	…	Factor 7	Weight
Characteristic root (post-rotation)	2.4890	…	0.001	
Variance explanation rate	35.56%	…	0.01%	
*Sa*	0.0312	…	0.1045	11.20%
*Sq*	0.0460	…	0.0881	12.16%
*Sbi*	0.5321	…	0.8577	15.69%
*Sci*	0.4526	…	0.2439	16.66%
*Svi*	0.2928	…	0.0099	15.33%
*Sku*	0.6047	…	0.4313	14.41%
Sck	0.2401	…	0.0041	14.56%

## Data Availability

The data presented in this study are available from the corresponding author upon reasonable request.
